# Vitamin D Counteracts *Mycobacterium tuberculosis*-Induced Cathelicidin Downregulation in Dendritic Cells and Allows Th1 Differentiation and IFNγ Secretion

**DOI:** 10.3389/fimmu.2017.00656

**Published:** 2017-05-31

**Authors:** Anna K. O. Rode, Martin Kongsbak, Marie M. Hansen, Daniel Villalba Lopez, Trine B. Levring, Anders Woetmann, Niels Ødum, Charlotte M. Bonefeld, Carsten Geisler

**Affiliations:** ^1^Faculty of Health and Medical Sciences, Department of Immunology and Microbiology, University of Copenhagen, Copenhagen, Denmark

**Keywords:** vitamin D, tuberculosis, T cells, dendritic cells, Th1, IFNγ, cathelicidin

## Abstract

Tuberculosis (TB) presents a serious health problem with approximately one-third of the world’s population infected with *Mycobacterium tuberculosis* in a latent state. Experience from the pre-antibiotic era and more recent clinical studies have established a beneficial role of sunlight and vitamin D in patients with TB. At the same time, experimental data have shown that Th1 cells through production of IFNγ are crucial for cathelicidin release by macrophages, bacterial killing, and containment of *M. tuberculosis* in granulomas. Paradoxically, vitamin D has repeatedly been ascribed an immune-suppressive function inhibiting Th1 differentiation and production of IFNγ in T cells. The aim of this study was to investigate this apparent paradox. We studied naïve human CD4^+^ T cells activated either with CD3 and CD28 antibodies or with allogeneic dendritic cells (DC) stimulated with heat-killed *M. tuberculosis* (HKMT) or purified toll-like receptor (TLR) ligands. We show that vitamin D does not block differentiation of human CD4^+^ T cells to Th1 cells and that interleukin (IL)-12 partially counteracts vitamin D-mediated inhibition of IFNγ production promoting production of equal amounts of IFNγ in Th1 cells in the presence of vitamin D as in T cells activated in the absence of vitamin D and IL-12. Furthermore, we show that HKMT and TLR2 ligands strongly downregulate cathelicidin expression in DC and that vitamin D counteracts this by upregulating cathelicidin expression. In conclusion, we demonstrate that vitamin D counteracts *M. tuberculosis*-induced cathelicidin downregulation and allows Th1 differentiation and IFNγ secretion.

## Introduction

Tuberculosis (TB) presents a serious health problem with more than 10 million new cases of active TB responsible for 1.8 million deaths in 2015 (1). The disease is caused by the pathogen *Mycobacterium tuberculosis*. It is presumed that approximately one-third of the world’s population is infected with *M. tuberculosis* in an asymptomatic latent state and that 5–10% of infected individuals develops active TB at some point in their lives either shortly after initial infection or as a progression from a latent infection (2). Children and individuals with an impaired immune system generally have a higher risk of developing active TB (3).

*M. tuberculosis* is transmitted through aerosol droplets to the lung alveoli, where the pathogen infects alveolar macrophages and subsequently dendritic cells (DC), neutrophils, and macrophages in the lung interstitium (4). Activation of naïve TB-specific T cells depends on the migration of infected DC from the lungs to the mediastinal lymph nodes (5–7). DC present *M. tuberculosis* antigens for the T cells and dependent on the cytokines present during the T cell receptor-mediated activation process, the CD4^+^ T helper (Th) cells differentiate to different types of effector cells in the lymph nodes (8, 9). Macrophages and DC become activated by stimulation through the toll-like receptors (TLR)2, 4, and 9 in response to *M. tuberculosis* and start production of pro-inflammatory cytokines such as tumor necrosis factor α (TNFα), interleukin (IL)-1β, and IL-12 (10). IL-12 plays a major role in the differentiation of Th cells to interferon γ (IFNγ)-producing Th1 effector cells by signaling through the IL-12 receptor (IL-12R), which results in phosphorylation and dimerization of signal transducer and activator of transcription (STAT)4. STAT4 then translocates to the nucleus and binds to regulatory elements of target genes, including the Th1 master transcription factor *TBX21, IL12R*β*2*, and *IFNG*. IFNγ induces phosphorylation of STAT1 that further activates transcription of *TBX21* and *IL12R*β*2*, which in turn act in a positive-feedback loop to amplify Th1 differentiation (8, 9). The resulting Th1 effector cells migrate out of the lymph nodes and are then recruited to the sites of infection.

In the latent state, *M. tuberculosis* is contained by the immune system in granulomas consisting mainly of infected macrophages surrounded by IFNγ-producing Th1 cells (2, 11). The importance of IFNγ in the immune response to mycobacteria is well established in both humans and mice. Thus, individuals with a mutation in genes related to the production of or response to IFNγ have a high susceptibility to mycobacterial infection (12–16). Likewise, mice that lack IFNγ or the IFNγ receptor are extremely susceptible to TB (17, 18). IFNγ is crucial for activation of macrophages and the formation and containment of *M. tuberculosis* in granulomas (13, 18). A central role of IFNγ is to enhance the ability of macrophages to kill intracellular pathogens such as *M. tuberculosis* (19, 20). The antimicrobial peptide cathelicidin plays an important role in the ability of macrophages to kill bacteria, and it has been reported that IFNγ increases the expression of cathelicidin in human monocytes and macrophages (21–23). Interestingly, these studies found that vitamin D is required for IFNγ-mediated enhancement of cathelicidin. This is in good agreement with several studies showing that vitamin D deficiency is associated with impaired expression of cathelicidin and increased susceptibility to infectious diseases, including TB (24–30), and it could be an important mechanism explaining the beneficial role of vitamin D in TB prevention and treatment (31, 32). In sharp contrast to this, several studies have shown that vitamin D inhibits the production of IFNγ in T cells (33–46). This creates a significant paradox in which vitamin D is required for efficient innate immune responses against *M. tuberculosis* but at the same time impairs Th1-mediated immune responses against *M. tuberculosis*.

The aim of this study was to look deeper into this apparent paradox and to determine whether vitamin D actually impedes Th1 differentiation and how vitamin D affects IFNγ production in Th1 cells and cathelicidin production in DC.

## Materials and Methods

### Chemicals

25(OH)D_3_ (BML-DM-100-0001) and 1,25(OH)_2_D_3_ (BML-DM200-0050) were from Enzo Life Sciences, Inc., Ann Arbor, MI, USA. Stock solutions of 2.5 mM 25(OH)D_3_ and 2.4 mM 1,25(OH)_2_D_3_ were prepared in anhydrous (≥99.5%) ethanol and stored at −20°C. To determine 1,25(OH)_2_D_3_ in the supernatants, we used the 1,25-dihydroxy vitamin D EIA kit (AC-62F1) from IDS, Tyne and Wear, UK according to the manufacturer’s instructions.

### Cell Culture

Mononuclear cells from blood were isolated by Lymphoprep (Axis-Shield, Oslo, Norway) density-gradient centrifugation using SepMate™ tubes (86460, Stemcell Technologies, Grenoble, France) from healthy donors after obtaining informed, written consent in accordance with the Declarations of Helsinki principles for research involving human objects. The study was approved by The Committees of Biomedical Research Ethics for the Capital Region in Denmark (H-16033682). Naïve CD4^+^ T cells were isolated and cultured as previously described (47). The purified naïve CD4^+^ T cells were cultured in serum-free X-VIVO 15 medium (BE02-060F, Lonza, Verviers, Belgium) at 37°C, 5% CO_2_ at a cell concentration of 1 × 10^6^ cells/ml in flat-bottomed 24-well tissue culture plates (142475) from Nunc and stimulated with Dynabeads human T-activator CD3/CD28 beads (111.31D, Life Technologies, Grand Island, NY, USA) at a cell to bead ratio of 5:2 for up to 3 days. Cells present in the culture after 3 days were defined as activated T cells. In some experiments, 25(OH)D_3_, 1,25(OH)_2_D_3_, or recombinant human IL-12 (219-IL, R&D Systems) was added at the indicated concentrations to the medium during the stimulation period. In Th1 polarization studies, purified naïve CD4^+^ T cells were cultured and stimulated as described above in the presence of recombinant human IL-12 (10 ng/ml) plus human IL-4 antibody (4 µg/ml, MAB204, R&D Systems). In some experiments, cells were counted after 72 h of activation using the automated cell counter NucleoCounter^®^ NC-100™ from Chemometec.

Monocytes used for generation of monocyte-derived DC were isolated from blood mononuclear cells using EasySep Human Monocyte Enrichment Kit (19059, Stemcell Technologies) according to the manufacturer’s protocol. Following isolation, monocytes were cultured in flat-bottomed six-well tissue culture plates (140675, Nunc) at a cell concentration of 5 × 10^5^ cells/ml for 6 days in medium (RPMI-1640, R5886, Sigma-Aldrich) with penicillin, streptomycin, l-glutamine, and 10% heat-inactivated FBS (10082-147, Gibco) supplemented with GM-CSF and IL-4 (both 50 ng/ml, AF-HDC, PeproTech) with a re-supplementation of medium and cytokines on day 3. On day 5, the differentiated DCs were supplemented with GM-CSF (50 ng/ml) and treated with either heat-killed *M. tuberculosis* (HKMT) (10 µg/ml, tlrl-hkmt, InvivoGen), Pam3CSK4 (300 ng/ml, tlrl-pms, Invivogen) as TLR2 ligand, lipopolysaccharides (LPS) from *E. coli* (50 ng/ml, L 5668, Sigma) as TLR4 ligand, or left untreated for 24 h, washed, and resuspended in X-VIVO 15 medium for use in mono- and co-cultures or in RPMI-1640 medium for use in 1,25(OH)_2_D_3_ titration experiments. For monocultures, 2.5–5 × 10^5^ cells/ml DC were plated in flat-bottomed 24-well tissue culture plates for 24 h in the presence or absence of 25(OH)D_3_ and with or without recombinant human IFNγ (R&D Systems) or with increasing concentrations of 1,25(OH)_2_D_3_ for titration studies. For co-culture studies, naïve human CD4^+^ T cells were purified from a different donor as described above and co-cultured with 1 × 10^5^ cells/ml DC in a ratio of 1:10 DC to T cells in flat-bottomed 24-well tissue culture plates for another 6 days in X-VIVO 15 medium.

### ELISA

IFNγ and IL-13 concentrations in the supernatants were determined by ELISA according to the manufacturer’s protocol (Ready-Set-Go; eBioscience).

### QPCR

mRNA expression was measured by real-time RT-PCR (QPCR). For this, cells were lysed in TRI reagent (T9424, Sigma-Aldrich) followed by addition of 1-bromo-3-chloropropane (B9673, Sigma Aldrich) to separate the sample into an aqueous and an organic phase. The RNA was precipitated from the aqueous phase using isopropanol supplemented with glycogen (10814-010, Invitrogen), washed with ethanol, and dissolved in RNase free water. Equal amounts of total RNA were used for complementary DNA (cDNA) synthesis using the high-capacity RNA-to-cDNA™ Kit from Applied Biosystems (4387406) according to manufacturer’s protocol. For QPCR, 12.5 ng cDNA was mixed with TaqMan^®^ Universal Master Mix II with UNG (4440038, Applied Biosystems), the target primer, and the eukaryotic 18 S rRNA primer (1509311, Applied Biosystems). The samples were run on a Stratagene Mx3000P™ real-time PCR machine (Agilent Technologies). The thermal profile was set to 2 min at 50°C, a 10 min hot start at 95°C, followed by cycles of 15 s at 95°C and 1 min at 60°C. Signal intensity was measured at the end of the 60°C step, and the threshold cycle values were related to eukaryotic 18S rRNA. The following primers were used; IFNG (Hs00989291_m1), CYP27B1 (Hs01096154_m1), vitamin D receptor (VDR) (Hs01045840_m1), CYP24A1 (Hs00167999_m1), TBX21 (Hs00203436_m1), IL-12RB2 (Hs00155486_m1), CXCL9 (Hs00171065_m1), CXCL10 (Hs00171042_m1), and CAMP (cathelicidin antimicrobial peptide) (Hs00189038_m1) all from Applied Biosystems.

### Western Blot Analysis

For Western blot analysis, whole cell lysates were obtained by treatment of the cells with lysis buffer (50 mM Tris pH 7.5, 150 mM NaCl, 1 mM MgCl_2_) supplemented with 1% Triton X-100, 1× Protease inhibitor cocktail (5872S, Cell Signaling Technology, Beverly, MA, USA) and 5 mM EDTA. The samples were run under reducing conditions on polyacrylamide gels for 2 h at 120 volt in 1× NuPAGE MOPS SDS Running buffer (K855, Amresco, Solon, OH, USA) in an XCell SureLock^®^ Mini-Cell Module from Life Technologies. The proteins were transferred to nitrocellulose membrane sheets (LC2001, Life Technologies) in 1× NuPAGE Transfer buffer (NP0006-1, Life Technologies) supplemented with 10% ethanol for 60 min at 50 V in an XCell II™ Blot Module from Life Technologies. The membranes were subsequently blocked for 60 min in Tris-buffered saline supplemented with 5% milk powder (70166, Sigma-Aldrich) and 0.1% Tween 20 (P1379, Sigma-Aldrich) and incubated at 4°C for 24 h with primary antibodies diluted in Tris-buffered saline supplemented with 5% bovine serum albumin (A4503, Sigma-Aldrich) and 0.1% Tween 20. The membranes were washed and the proteins visualized following 60 min incubation at room temperature with secondary HRP-rabbit anti-mouse Ig and HRP-swine anti-rabbit Ig using ECL (RPN2232, Sigma Aldrich) technology. The anti-phospho-STAT1 (9171) and phospho-STAT4 (5267) antibodies were from Cell Signaling Technologies, the anti-STAT1 (SC-346), STAT4 (SC-486), and VDR (SC-13133) antibodies were from Santa Cruz Biotechnology, Santa Cruz, CA, USA and the anti-GAPDH (Ab9485) was from Abcam, Cambridge, UK. For band density quantification, ECL-exposed sheets were analyzed in a ChemiDoc MP Imaging System from Bio-Rad.

### Statistical Analysis

Data are shown as mean ± SEM. Statistical analyses were performed using Student’s *t*-test with a 5% significance level, paired observations, and equal variance. * indicates *p* ≤ 0.05.

## Results

### IL-12 Partially Counteracts Vitamin D-Mediated Inhibition of IFNγ Production in CD4^+^ T Cells

**Figure 1:**
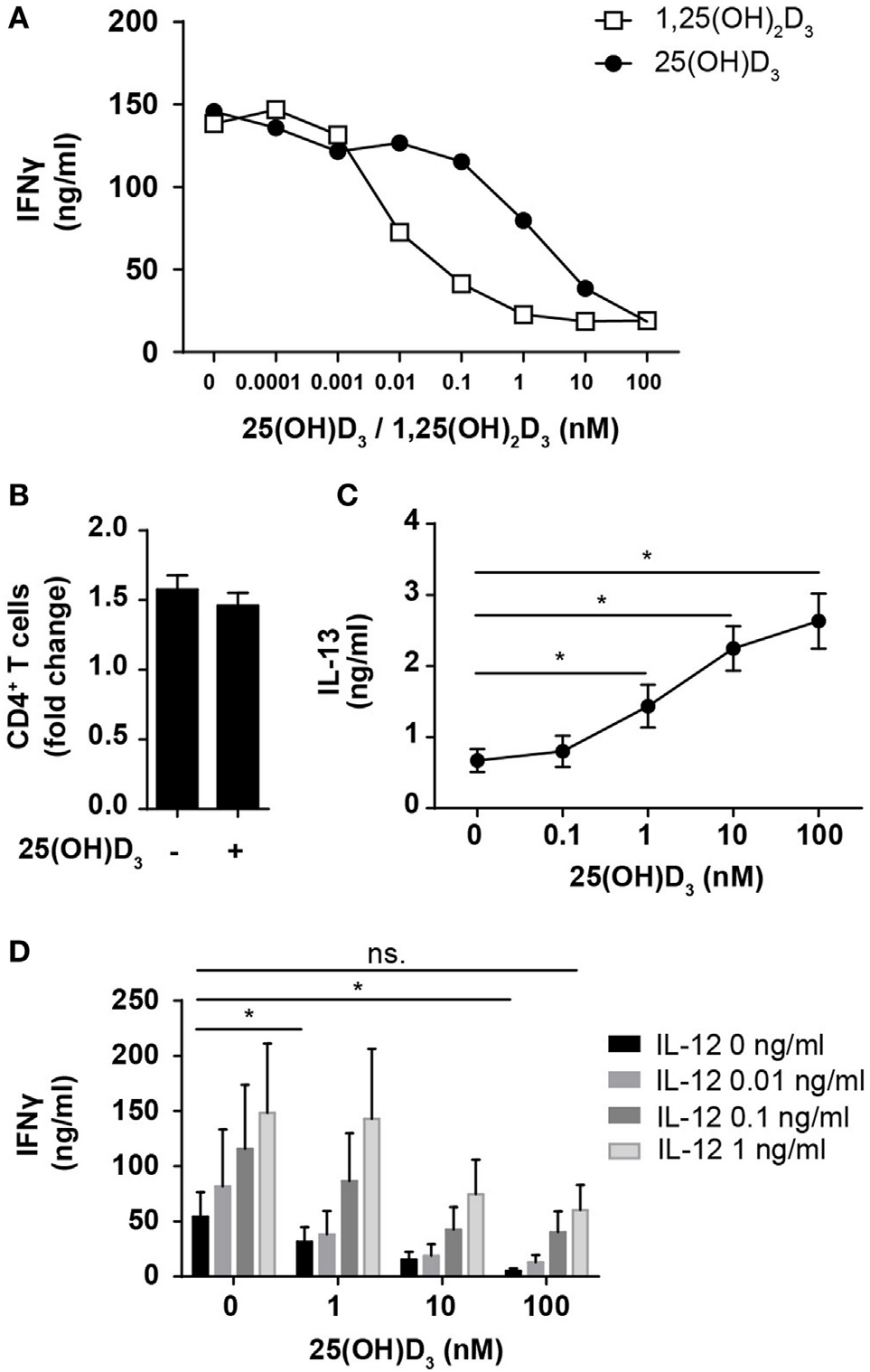
IL-12 counteracts vitamin D-mediated inhibition of IFNγ production in CD4^+^ T cells. **(A)** IFNγ concentration in the supernatants of T cells activated for 72 h in the presence of the indicated concentrations of 25(OH)D_3_ or 1,25(OH)_2_D_3_. Representative graphs from two independent experiments with three donors are shown. **(B)** Number of CD4^+^ T cells in the cell cultures after activation for 72 h in the presence or absence of 100 nM 25(OH)D_3_ relative to initial cell number (mean + SEM, *n* = 12). **(C)** IL-13 concentration in the supernatant of T cells activated for 72 h in the presence of the indicated concentrations of 25(OH)D_3_ (mean ± SEM, *n* = 6). **(D)** IFNγ concentration in the supernatants of T cells activated for 72 h in the presence of the indicated concentrations of 25(OH)D_3_ and IL-12 (mean + SEM, *n* ≥ 4).

Most previous studies on the effect of vitamin D on IFNγ production in human T cells have been performed using heterologous cell populations or purified CD4^+^ T cells in cell culture medium supplemented with serum and non-physiological, high concentrations (1–100 nM) of the active form of vitamin D (1,25(OH)_2_D) (41–45). Various sources of serum contain different concentrations of the inactive form of vitamin D (25(OH)D), 1,25(OH)_2_D, and the vitamin D-binding protein, all of great importance when investigating the effect of vitamin D on T cells (24, 46). To study the direct effect of 25(OH)D_3_ and 1,25(OH)_2_D_3_ on IFNγ production in human Th cells under strictly defined conditions, we isolated naïve CD4^+^ T cells and stimulated them with human T-activator CD3/CD28 beads in serum-free medium in the absence or presence of 25(OH)D_3_ or 1,25(OH)_2_D_3_. We found that 1,25(OH)_2_D_3_ strongly inhibited IFNγ production (~15-fold) at physiological concentrations (60–110 pM) (Figure 1A). Furthermore, we found that 25(OH)D_3_ at physiological concentrations (50–125 nM) inhibited IFNγ production to the same degree as 1,25(OH)_2_D_3_ (Figure 1A), which confirms that T cells have the ability to convert the inactive 25(OH)D_3_ to the active 1,25(OH)_2_D_3_ (46, 47). It is well known that anti-CD3/CD28 bead stimulation promotes expansion of CD4^+^ T cells (48). To investigate whether the lower levels of IFNγ in vitamin D-treated cells could be attributed to fewer cells, we stimulated CD4^+^ T cells for 72 h in the presence or absence of 100 nM 25(OH)D_3_ and subsequently counted the cells using an automated cell counter. At this time point, we found that there were approximately 1.5-fold more CD4^+^ T cells in the cultures compared to the initial cell number independently of the absence or presence of vitamin D (Figure 1B). To exclude a general toxic effect of vitamin D on the T cells, we subsequently measured secretion of IL-13 in parallel with IFNγ. We found that vitamin D increased IL-13 secretion in parallel with its inhibition of IFNγ secretion (Figure 1C). Thus, the decreased levels of IFNγ were not caused by a reduced cell number or a toxic effect of vitamin D but most likely by a specific effect of vitamin D on IFNγ secretion. Previous studies have reported that 1,25(OH)_2_D_3_ inhibits production of IFNγ and augment the production of IL-4, and based on these observations it has been concluded that vitamin D restrains Th1 differentiation and promotes Th2 differentiation (41–45). However, to our knowledge, the effect of physiological concentrations of vitamin D on IFNγ production in T cells activated under Th1-inducing conditions has not been reported. To study this, we activated naïve CD4^+^ T cells in the absence or presence of IL-12 and 25(OH)D_3_. In the absence of 25(OH)D_3_, IL-12 increased IFNγ production (Figure 1D). Interestingly, although 25(OH)D_3_ at physiological concentrations did reduce IFNγ production in IL-12 treated cells, these cells still produced equal amounts of IFNγ as T cells activated in the absence of 25(OH)D_3_ and IL-12 (Figure 1D). From these experiments, we could conclude that 1,25(OH)_2_D_3_ inhibits the production of IFNγ in CD4^+^ effector T cells but that IL-12 partially rescues IFNγ production in the presence of vitamin D allowing production of similar amounts of IFNγ as in T cells activated in the absence of vitamin D and IL-12.

### IL-12 Does Not Inhibit 1,25(OH)_2_D_3_ Production or VDR Expression and Function in CD4^+^ T Cells

**Figure 2:**
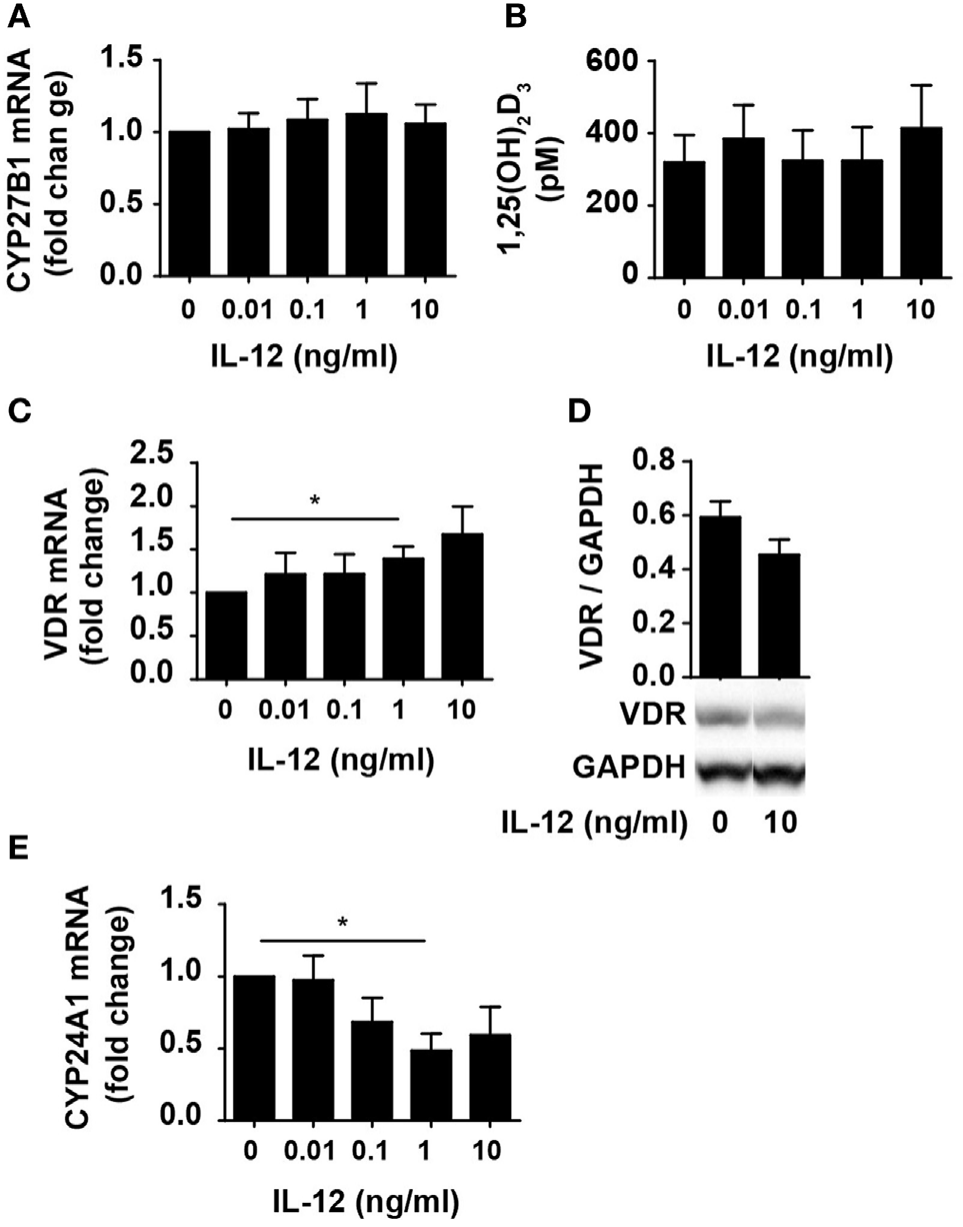
IL-12 does not inhibit 1,25(OH)_2_D_3_ production or VDR expression and function in CD4^+^ T cells. Relative CYP27B1 **(A)**, VDR **(C)**, and CYP24A1 **(E)** expression in T cells activated for 72 h in the presence of 100 nM 25(OH)D_3_ and the indicated concentration of IL-12. Data are normalized to activated T cells incubated with 100 nM 25(OH)D_3_ in the absence of IL-12 (mean + SEM, *n* ≥ 6). **(B)** 1,25(OH)_2_D_3_ production in T cells activated for 72 h in the presence of 100 nM 25(OH)D_3_ and the indicated concentration of IL-12 (mean + SEM, *n* ≥ 4). **(D)** Representative Western blot (lower panel) and quantification (upper panel) of VDR with GAPDH as loading control from T cells activated for 72 h in the presence of 100 nM 25(OH)D_3_ and in the presence or absence of 10 ng/ml IL-12 (mean + SEM, *n* = 4). Western blots including protein ladder are shown in the Figure S1 in Supplementary Material.

One way for IL-12 to counteract vitamin D-mediated inhibition of IFNγ production in CD4^+^ T cells could be by limiting the ability of the cells to convert 25(OH)D_3_ to 1,25(OH)_2_D_3_. To investigate this, we studied whether IL-12 affected the expression of CYP27B1, the enzyme responsible for converting 25(OH)D_3_ to 1,25(OH)_2_D_3_. We activated naïve CD4^+^ T cells in the presence of 100 nM 25(OH)D_3_ in the absence or presence of IL-12 and measured the expression levels of CYP27B1 mRNA in the cells. We found that IL-12 did not influence the expression of CYP27B1 (Figure 2A). Furthermore, we directly measured the production of 1,25(OH)_2_D_3_ in the supernatants and found that IL-12 did not affect the production of 1,25(OH)_2_D_3_ (Figure 2B). Another way IL-12 might counteract vitamin D-mediated inhibition of IFNγ production in CD4^+^ T cells could be by inhibiting the expression or function of the VDR. However, we found that IL-12 slightly increased VDR expression on the mRNA level (Figure 2C). To further investigate whether IL-12 affected VDR expression, we determined the effect of IL-12 on VDR protein levels. We did not observe any significant effect of IL-12 on VDR expression at the protein level (Figure 2D). CYP24A1 is the enzyme that initiates degradation of 1,25(OH)_2_D_3_, and CYP24A1 expression is dependent on functional 1,25(OH)_2_D_3_/VDR complexes. Consequently, we measured the CYP24A1 levels in cells treated with or without IL-12. We found that the cells produced very high amounts of CYP24A1 in the presence of 100 nM 25(OH)D_3_ (approximately 100.000-fold upregulated compared to unstimulated T cells) although IL-12 slightly reduced CYP24A1 expression (Figure 2E). Taken together, these experiments indicated that IL-12 does not counteract vitamin D-mediated inhibition of IFNγ production in CD4^+^ T cells by reducing their ability to produce 1,25(OH)_2_D_3_ or by reducing their VDR expression or function.

### Vitamin D Does Not Prevent Th1 Differentiation

**Figure 3:**
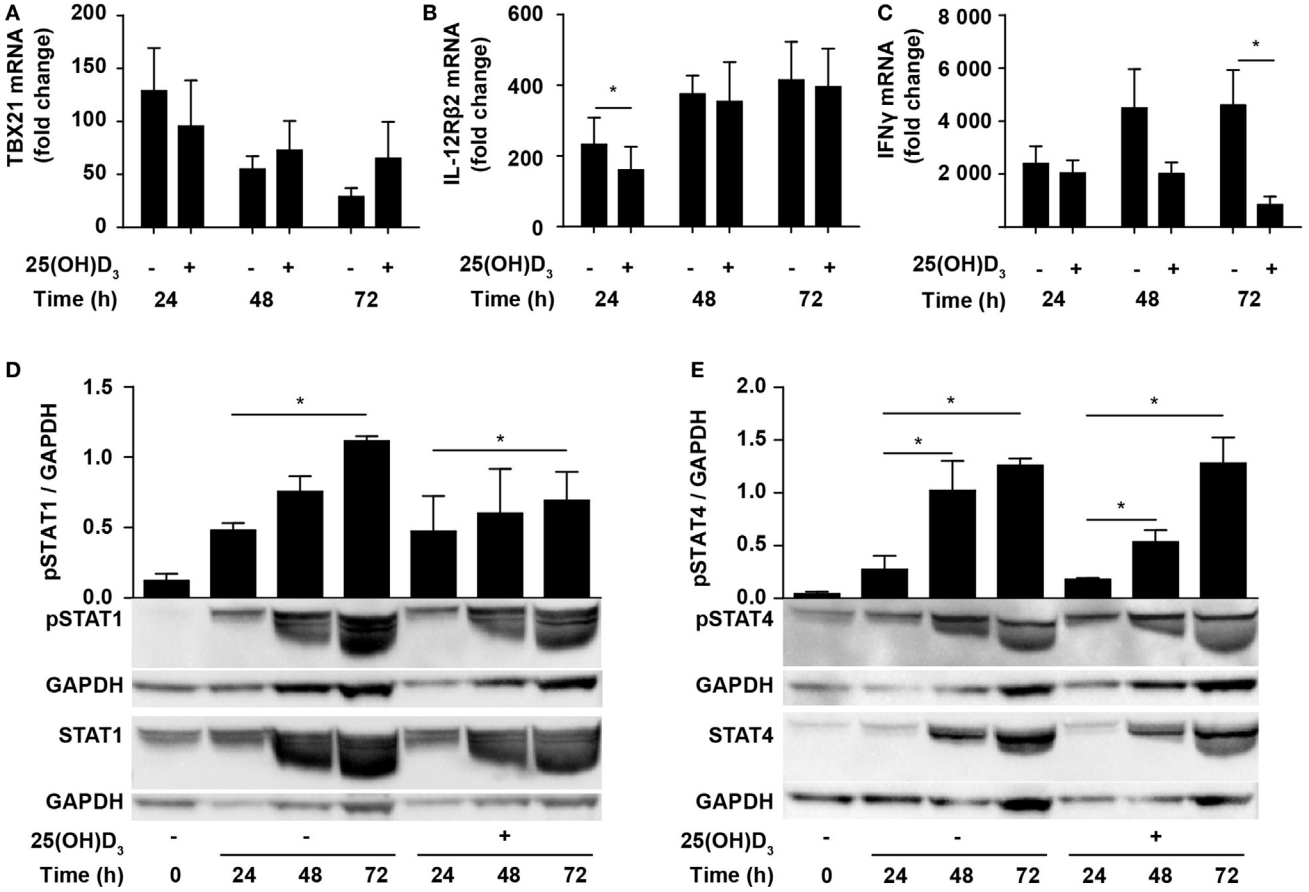
Vitamin D does not prevent Th1 differentiation. Relative TBX21 **(A)**, IL-12Rβ2 **(B)**, and IFNγ **(C)** expression in T cells activated for 24, 48, or 72 h in Th1-polarizing medium in the presence or absence of 100 nM 25(OH)D_3_. Data are normalized to unstimulated T cells (mean + SEM, *n* = 6). Representative Western blots (lower panel) and quantification (upper panel) of phosphorylated STAT1 and total STAT1 **(D)** and phosphorylated STAT4 and total STAT4 **(E)** with GAPDH as loading control from T cells activated for 0, 24, 48, and 72 h in Th1-polarizing medium in the presence or absence of 100 nM 25(OH)D_3_. Western blots including protein ladder are shown in the Figure S2 in Supplementary Material.

Due to the inhibition of IFNγ production, it has been suggested that vitamin D impedes differentiation of Th1 cells. However, whether vitamin D actually inhibits Th1 differentiation is still not known. To directly study whether vitamin D affects Th1 differentiation, we stimulated naïve CD4^+^ T cells under classical Th1 conditions with IL-12 and anti-IL-4 in the absence or presence of 25(OH)D_3_ and measured the expression levels of TBX21 and IL-12Rβ2. Compared to naïve T cells, cells activated for 24 h in the absence of 25(OH)D_3_ upregulated TBX21 approximately 120-fold. Although not significantly, 25(OH)D_3_ slightly reduced TBX21 upregulation at 24 h but had no inhibitory effect on TBX21 expression after 48 and 72 h (Figure 3A). Likewise, IL-12Rβ2 expression was slightly reduced after 24 h but unaffected by 25(OH)D_3_ after 48 and 72 h of stimulation (Figure 3B). While vitamin D did not affect TBX21 and IL-12Rβ2 expression in cells stimulated for 48 and 72 h, it clearly reduced IFNγ expression in the same cells (Figure 3C). Thus, these experiments indicated that vitamin D does not block differentiation of Th1 cells but directly affects the transcription of the *IFNG* gene. Although reduced, IFNγ expression in Th1 cells treated with vitamin D was still highly upregulated (1000- to 2000-fold, Figure 3C) and they produced high levels of IFNγ (Figure 1D). In line with the strong production of IFNγ, we found that vitamin D did not affect STAT1 phosphorylation significantly during T cell activation (Figure 3D). Likewise, STAT4 phosphorylation was not significantly affected of vitamin D (Figure 3E).

**Figure 4:**
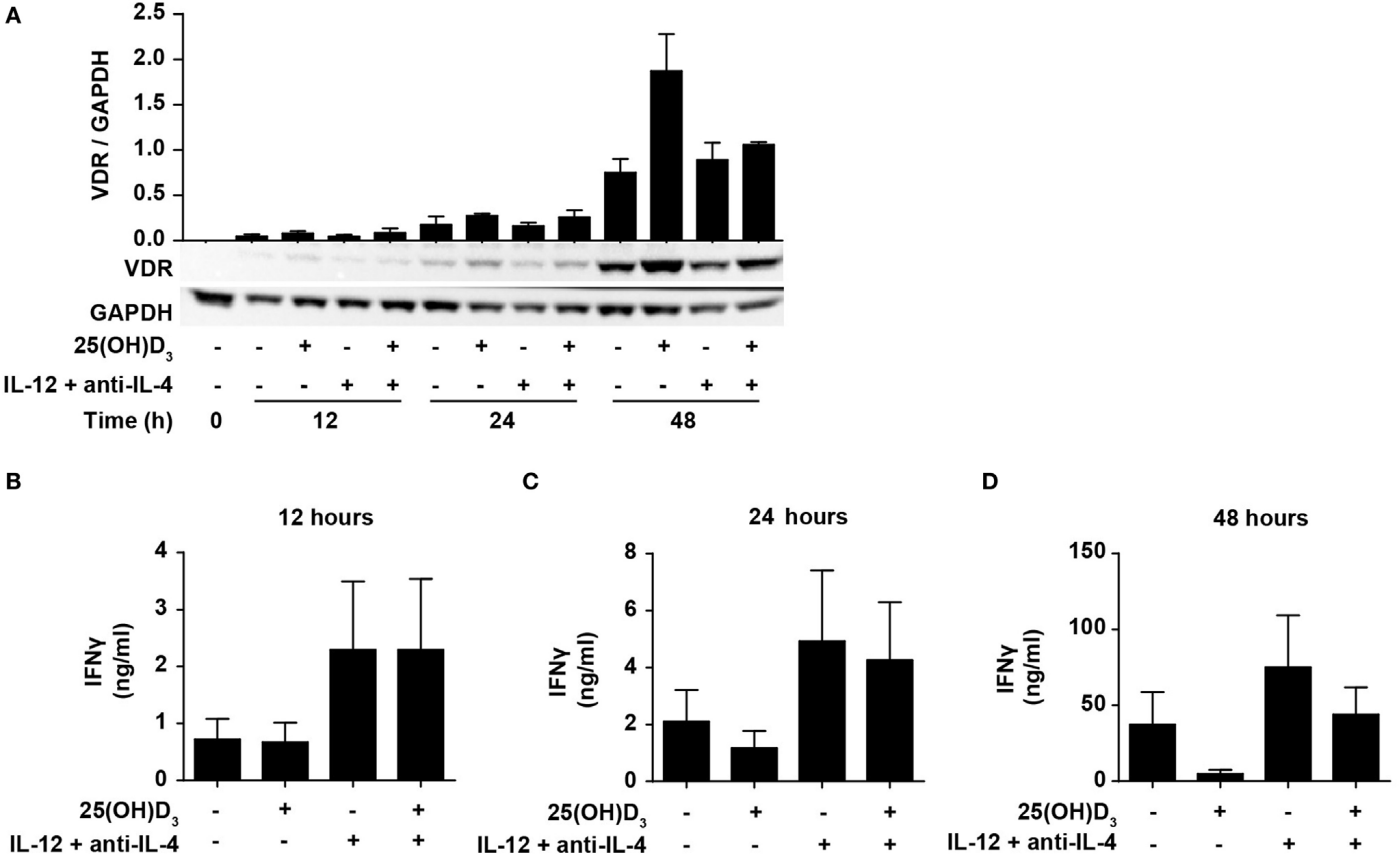
IL-12-mediated signaling is initiated before vitamin D signaling. **(A)** Representative Western blots (lower panel) and quantification (upper panel) of VDR with GAPDH as loading control from T cells activated for 0, 12, 24, and 48 h in the presence or absence of 100 nM 25(OH)D_3_ and Th1-polarizing medium (IL-12 + anti-IL-4) (mean + SEM, *n* = 2). IFNγ concentration in the supernatants of T cells activated for 12 h **(B)**, 24 h **(C)**, or 48 h **(D)** in the presence or absence of 100 nM 25(OH)D_3_ and Th1-polarizing medium (mean + SEM, *n* = 4). Western blots including protein ladder are shown in the Figure S3 in Supplementary Material.

IFNγ itself plays an important role during Th1 differentiation as STAT1 activated by IFNγ further activates transcription of *TBX21* and *IL12R*β*2* (8, 9). We have previously demonstrated that naïve human CD4^+^ T cells do not express the VDR but that they start to express it 24–48 h after TCR/CD28 stimulation (46, 49). This suggests that vitamin D cannot affect IFNγ production in the early stages of T cell activation due to the lack of VDR. To study the effect of 25(OH)D_3_ and Th1-inducing conditions on the early kinetics of VDR expression, we stimulated naïve CD4^+^ T cells for 12, 24, and 48 h in the presence or absence of 25(OH)D_3_ and Th1-inducing conditions. We could confirm that only very low levels of VDR are present after 12 and 24 h of stimulation, and that substantial VDR expression levels are found after 48 h of stimulation (Figure 4A). In agreement, we found that at 12 h of stimulation, vitamin D did not influence IFNγ production neither in cells activated in the absence or presence of Th1 conditions (Figure 4B). In contrast, Th1 conditions acted rapidly with increased production of IFNγ already 12 h after stimulation (Figure 4B). After 24 h of stimulation, a minor inhibitory effect of vitamin D on IFNγ production was seen (Figure 4C), and after 48 h vitamin D substantially inhibited IFNγ production (Figure 4D). These experiments indicated that IL-12-mediated signaling is initiated rapidly after initial T cell stimulation with increased IFNγ production as a consequence, and that this takes place before the VDR is expressed at sufficiently high levels to inhibit IFNγ production. These observations support that CD4^+^ T cells activated under Th1-inducing conditions start differentiation toward Th1 cells even in the presence of vitamin D.

### Vitamin D Is Required for Concomitant Production of IFNγ and Cathelicidin in DC–T Cell Co-Cultures

**Figure 5:**
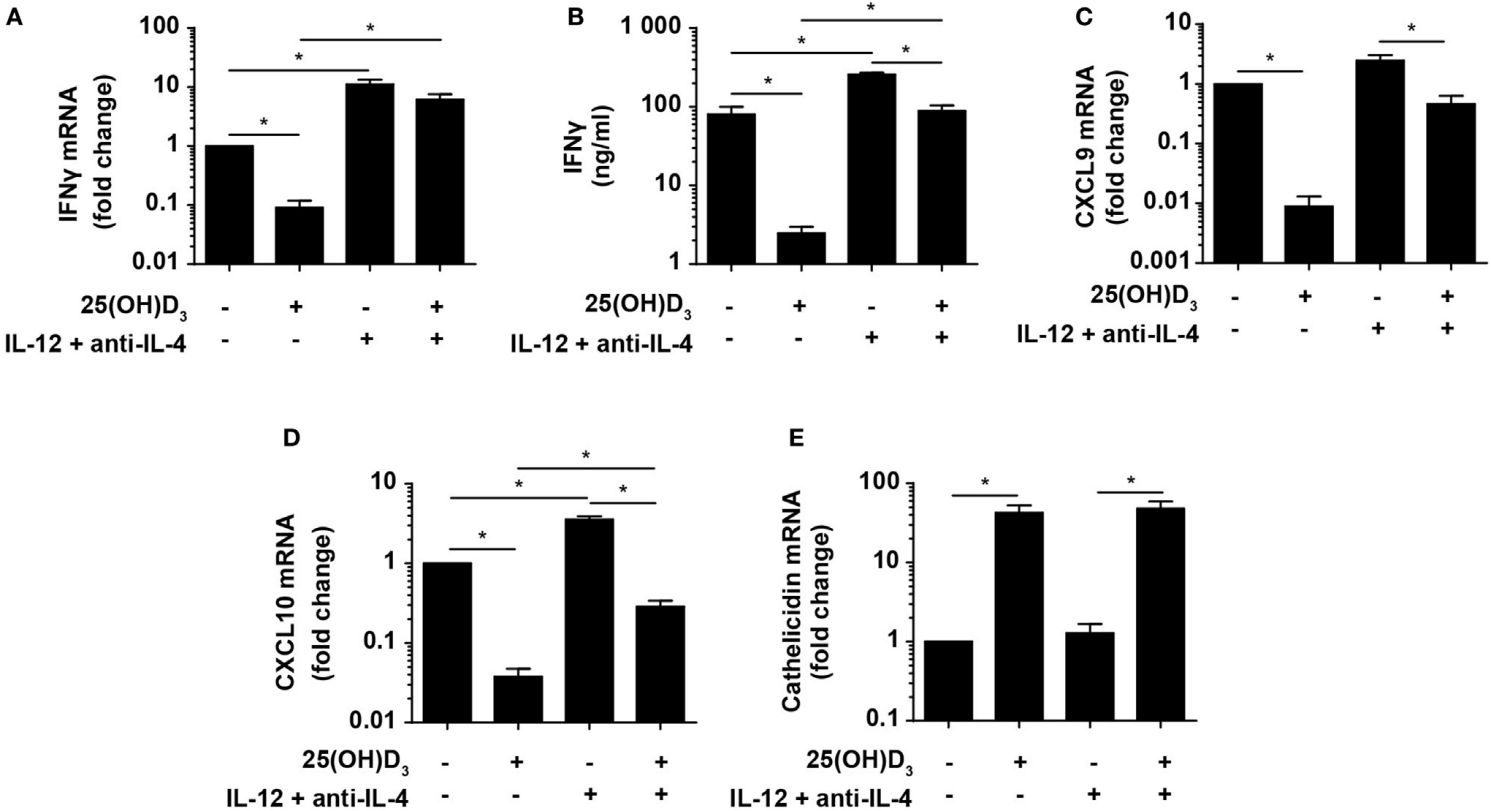
Vitamin D is required for concomitant production of IFNγ and cathelicidin in DC–T cell cultures. Relative expression **(A)** and production **(B)** of IFNγ and relative CXCL9 **(C)**, CXCL10 **(D)**, and cathelicidin **(E)** expression in DC–T cell co-cultures incubated in the presence or absence of 100 nM 25(OH)D_3_ and Th1-polarizing medium (IL-12 + anti-IL-4). Data are normalized to the values obtained from co-cultures incubated in the absence of 25(OH)D_3_ and Th1-polarizing medium (mean + SEM, *n* = 4).

Activation of naïve TB-specific T cells depends on the migration of infected DC from the lungs to the mediastinal lymph nodes where the DC present TB-derived antigens for the T cells (5–7). It has been suggested that antigen-presenting cells mediate the vitamin D-induced inhibition of IFNγ production in T cells (36, 38, 40), and the absence of antigen-presenting cells in our experimental set up could maybe explain why we observed IFNγ production in purified T cells stimulated in the presence of vitamin D and IL-12. To investigate this possibility, we cultured naïve CD4^+^ T cells with allogeneic DC for 6 days with or without 25(OH)D_3_ in the absence or presence of Th1-inducing conditions and subsequently measured IFNγ expression and production. Vitamin D strongly inhibited IFNγ mRNA expression (~15-fold) (Figure 5A) and production (~20-fold) (Figure 5B) in the absence of Th1-inducing conditions. Th1-inducing conditions increased IFNγ expression and production and counteracted the inhibitory effect of vitamin D. Thus, vitamin D only inhibited IFNγ mRNA expression ~2-fold and IFNγ production ~3-fold in the presence of Th1-inducing conditions (Figures 5A,B). Interestingly, IFNγ expression and production in cells cultured with vitamin D under Th1-inducing conditions were at least as high as in cells cultured without vitamin D and Th1-inducing conditions (Figures 5A,B). This indicated that DC do not shut down IFNγ production in T cells in the presence of vitamin D. Taken together, we could conclude that vitamin D strongly inhibits IFNγ expression and production in T cells in the absence of Th1-inducing conditions, and that Th1-inducing conditions partially rescue IFNγ production in the presence of vitamin D independently on the presence or absence of DC.

Cellular responses mediated by IFNγ are primarily caused by gene expression modulation. Primary IFNγ-responsive genes are induced early by binding of STAT1 dimers to gamma-activating sequences in the promoter of target genes such as IRF1, CXCL9, and CXCL10 (50). To study whether the levels of IFNγ produced in Th1 cells in the presence of vitamin D were sufficient to affect IFNγ-responsive genes, we measured the expression of CXCL9 and CXCL10 in the same co-cultures as described above. We found that vitamin D reduced CXCL9 and CXCL10 expression ~110 and ~25-fold in Th0 cells, respectively (Figures 5C,D). In contrast, vitamin D only reduced CXCL9 and CXCL10 expression ~2- and ~3-fold in Th1 cells compared to untreated Th0 cells. This demonstrated that the cellular responses to the levels of IFNγ produced in Th1 cells in the presence of vitamin D almost equaled the responses to the levels of IFNγ produced in Th0 cells in the absence of vitamin D.

As the importance of IFNγ in the immune response to mycobacteria is well established, it is puzzling why vitamin D, associated with a beneficial role in TB prevention and treatment, reduces IFNγ production. Cathelicidin plays an important role in the killing of especially intracellular pathogens such as *M. tuberculosis* in macrophages (51). Furthermore, it has been shown that cathelicidin expression is dependent on vitamin D, and that IFNγ increases the synthesis of cathelicidin in macrophages (21–23). To study how cathelicidin is regulated in DC, we cultured naïve CD4^+^ T cells with allogeneic DC for 6 days as described above with or without 25(OH)D_3_ in the absence or presence of Th1-inducing conditions and subsequently measured cathelicidin expression. We found that cathelicidin upregulation was absolutely dependent on vitamin D but independent of Th1-inducing conditions (Figure 5E). This indicated that vitamin D without IFNγ is sufficient to induce cathelicidin upregulation, and that IFNγ without vitamin D does not affect cathelicidin expression in DC.

### Vitamin D Counteracts the Inhibitory Effect of *M. tuberculosis* on Cathelicidin Expression in DC

**Figure 6:**
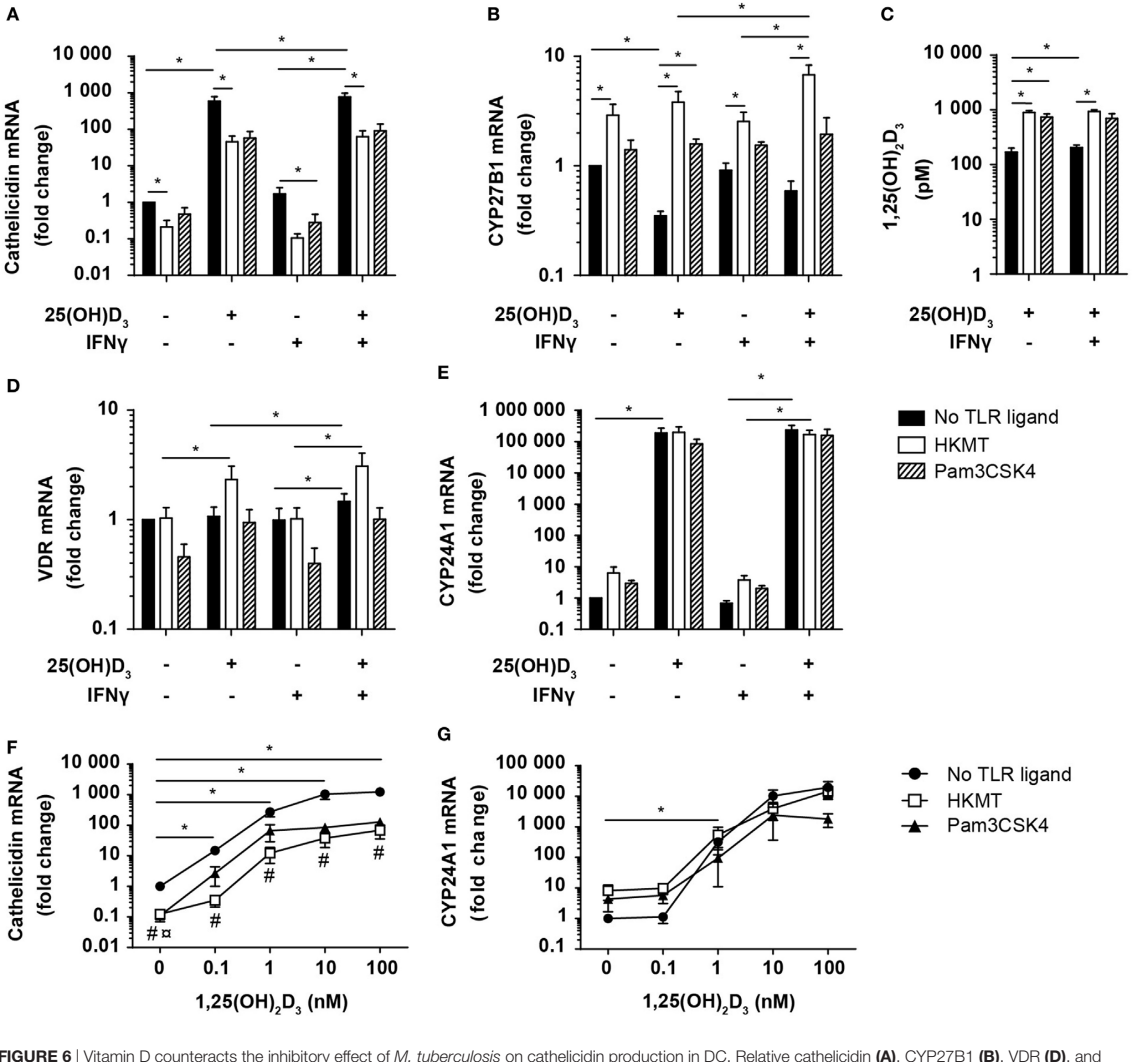
Vitamin D counteracts the inhibitory effect of *M. tuberculosis* on cathelicidin production in DC. Relative cathelicidin **(A)**, CYP27B1 **(B)**, VDR **(D)**, and CYP24A1 **(E)** expression and 1,25(OH)_2_D_3_ production **(C)** in untreated DC (no TLR ligand) or DC treated with HKMT or Pam3CSK4 and subsequently incubated for 24 h in the absence or presence of 100 nM 25(OH)D_3_ and 10 ng/ml IFNγ. Data are normalized to the values obtained from untreated DC in the absence of 25(OH)D_3_ and IFNγ (mean + SEM, *n* ≥ 3), *indicates *p* ≤ 0.05. Relative cathelicidin **(F)** and CYP24A1 **(G)** expression in untreated DC (no TLR ligand) or DC treated with HKMT or Pam3CSK4 incubated for 24 h with the indicated concentrations of 1,25(OH)_2_D_3_. Data are normalized to untreated DC in the absence of 1,25(OH)_2_D_3_ (mean ± SEM, *n* ≥ 3), * indicates *p* ≤ 0.05 for no TLR ligand untreated versus 1,25(OH)_2_D_3_ treated, # indicates *p* ≤ 0.05 for HKMT versus no TLR ligand, ¤ indicates *p* ≤ 0.05 for Pam3CSK4 versus no TLR ligand.

Previous studies have demonstrated that IFNγ acts in synergy with TLR2 ligands to upregulate cathelicidin expression in human monocytes and macrophages (21, 22). To determine how vitamin D, IFNγ, and HKMT/TLR2 ligands affect cathelicidin expression in DC, we incubated DC in the absence or presence of 25(OH)D_3_, IFNγ, and HKMT or Pam3CSK4, and measured the expression of cathelicidin. Surprisingly, we found that HKMT and Pam3CSK4 significantly downregulated cathelicidin expression both in the absence and presence of vitamin D (Figure 6A). In contrast, vitamin D strongly upregulated cathelicidin expression both in the absence and presence of HKMT or Pam3CSK4. Although HKMT and Pam3CSK4 downregulated cathelicidin expression ~13- and ~10-fold in the presence of vitamin D, respectively, the expression of cathelicidin was still ~215- and ~120-fold higher in DC stimulated with HKMT/Pam3CSK4 in the presence of vitamin D than in the absence of vitamin D (Figure 6A). IFNγ by itself did not affect cathelicidin expression.

To determine whether the HKMT/Pam3CSK4-induced inhibition of cathelicidin expression was caused by a reduced ability to produce or respond to 1,25(OH)_2_D_3_, we incubated DC in the absence or presence of 25(OH)D_3_, IFNγ, and HKMT/Pam3CSK4 and measured the expression of CYP27B1, VDR, and CYP24A1, and the production of 1,25(OH)_2_D_3_. We found that HKMT/Pam3CSK4 increased CYP27B1 expression, especially in the presence of 25(OH)D_3_ (Figure 6B). In accordance, although untreated DC produced high amounts of 1,25(OH)_2_D_3_, HKMT/Pam3CSK4 increased the 1,25(OH)_2_D_3_ production four- to fivefold (Figure 6C). In addition, HKMT slightly increased VDR expression in the presence of 25(OH)D_3_ (Figure 6D). This suggested that HKMT/Pam3CSK4-treated DC should be at least as efficient as untreated DC to respond to 1,25(OH)_2_D_3_. This was supported by the expression of comparable levels of the 1,25(OH)_2_D_3_/VDR-dependent CYP24A1 in DC treated or untreated with HKMT/Pam3CSK4 in the presence of 25(OH)D_3_ (Figure 6E). These observations indicated that the inhibitory effect of HKMT/Pam3CSK4 on cathelicidin expression was caused by a vitamin D/VDR-independent mechanism. To test this hypothesis, we incubated DC in the absence or presence of HKMT or Pam3CSK4 and increasing concentrations of 1,25(OH)_2_D_3_. In all concentrations of 1,25(OH)_2_D_3_ tested, we found that HKMT/Pam3CSK4 inhibited cathelicidin expression (Figure 6F). Interestingly, stimulation with the more complex HKMT resulted in a ~23-fold reduction in cathelicidin expression, whereas the pure TLR2 ligand Pam3CSK4 caused an ~8-fold reduction in cathelicidin expression at all concentrations of 1,25(OH)_2_D_3_ tested. This suggested that HKMT affected the DC through additional receptors than TLR2. In agreement, we found that the TLR4 ligand LPS also inhibited cathelicidin expression in DC (data not shown). In contrast to the effect on cathelicidin expression, HKMT/Pam3CSK4 did not affect CYP24A1 expression, supporting that HKMT/Pam3CSK4 did not inhibit the ability of DC to respond to 1,25(OH)_2_D_3_/VDR complexes (Figure 6G). Taken together, these data indicated that *M. tuberculosis* strongly inhibits cathelicidin expression in DC through TLR signaling but that vitamin D increases cathelicidin expression and thereby more than overcome the inhibitory effect of *M. tuberculosis*.

## Discussion

In this study, we show that vitamin D does not block differentiation of naïve human CD4^+^ T cells to Th1 cells and that IL-12 partially rescues IFNγ production in T cells exposed to vitamin D. Furthermore, we demonstrate that HKMT/TLR2 ligands strongly downregulate cathelicidin expression in DC and that vitamin D counteracts this by upregulating cathelicidin expression.

Since the first studies on the effect of vitamin D on IFNγ production in human T cells (33, 34) several studies have confirmed that vitamin D inhibits IFNγ production in both human and mouse T cells (35–46). Some studies found that this was an indirect effect mediated via antigen-presenting cells (36, 38, 40), whereas others found a direct effect of vitamin D on T cells (41–43). We found that both the inactive and the active form of vitamin D inhibit IFNγ production in purified CD4^+^ T cells. Thus, our study confirms that activated T cells can convert inactive vitamin D to active vitamin D, and that vitamin D has a direct effect on human T cells. This is in good accordance with expression of both CYP27B1 and the VDR in activated human T cells (46, 47, 49, 52). Based on the impaired IFNγ production and increased IL-4 production in T cells exposed to vitamin D, it has been concluded that vitamin D restrains Th1 differentiation and promotes Th2 differentiation. However, only few studies have investigated whether vitamin D actually affects the master transcription factors TBX21 and GATA3 regulating Th1 and Th2 differentiation. One study found that although 1,25(OH)_2_D_3_ inhibited IFNγ production, it had no effect on the levels of TBX21 and GATA3 in mouse T cells activated under Th0, Th1, or Th2 conditions (37). Another study found a very discrete increase in GATA3 expression in mouse T cells activated in the presence of 1,25(OH)_2_D_3_ under Th0 conditions, but they did not study TBX21 expression nor the effect of Th1 conditions (53). We found that vitamin D did not significantly affect the expression of TBX21 in human T cells activated under Th1 conditions. Likewise, although IL-12Rβ2 upregulation was slightly inhibited at 24 h, vitamin D did not affect IL-12Rβ2 expression at later time points. This indicated that vitamin D does not restrain Th1 differentiation. STAT4 and STAT1 signaling play critical roles in the differentiation of Th1 cells (8). We are not aware of previous studies that have investigated the effect of vitamin D on STAT1 and STAT4 activation in human T cells. In the present study, we found that vitamin D did not significantly affect phosphorylation of STAT1 and STAT4 in T cells activated under Th1 conditions, which further supports that vitamin D does not block differentiation of Th1 cells. Thus, vitamin D most likely directly affects transcription of the *IFNG* gene and not the differentiation of naïve CD4^+^ T cells to Th1 cells. This is in accordance with previous studies, which found that 1,25(OH)_2_D_3_/VDR complexes directly bind to the *IFNG* promoter and inhibit *IFNG* transcription (54) and that the fraction of IFNγ-producing T cells is not reduced in T cells activated in the presence of vitamin D under Th1 conditions (55). The kinetic studies illustrated in Figures 3 and 4 in this study also strongly support that vitamin D does not inhibit Th1 differentiation. The VDR is not or only weakly expressed in T cells for the first 24 h of stimulation, and consequently vitamin D cannot affect IFNγ expression or production at these early time points. In contrast, IL-12 signaling is efficient already shortly after T cell activation and augments IFNγ production and initiates Th1 differentiation.

It has been demonstrated that the promoter of the human cathelicidin gene contains a consensus vitamin D response element, and that cathelicidin is upregulated by vitamin D (56, 57). Shortly after these studies, it was found that signaling through TLR2 induced upregulation of CYP27B1 and the VDR in human monocytes and macrophages but not in DC (24). Furthermore, upregulation of cathelicidin and growth inhibition of *M. tuberculosis* were seen if the cell culture media was supplemented with 25(OH)D_3_ (24). A subsequent study confirmed that the vitamin D-mediated antimicrobial activity against *M. tuberculosis* in the human monocytic cell line THP-1 was dependent on cathelicidin (51). In accordance with Liu et al. (24), we found that HKMT/TLR2 triggering did not significantly affect VDR expression in DC. In contrast to VDR, we found that the TLR2 ligand Pam3CSK4 and especially HKMT upregulated CYP27B1 expression and 1,25(OH)_2_D_3_, production in the presence of 25(OH)D_3_. However, although HKMT/Pam3CSK4 upregulated CYP27B1, untreated DC still expressed high levels of CYP27B1. Accordingly, we found that even untreated DCs have a high capacity to convert 25(OH)D_3_ to 1,25(OH)_2_D_3_, and to respond to 1,25(OH)_2_D_3_ with strong upregulation of cathelicidin and CYP24A1 expression. In sharp contrast to studies finding that TLR2 ligands upregulated cathelicidin in human monocytes and macrophages in a vitamin D-dependent manner (21, 22, 24), we found that HKMT profoundly downregulated cathelicidin in human DC. Although HKMT is described as a primary TLR2 ligand, it has been reported that *M. tuberculosis* stimulates a range of other receptors, including TLR4 and 9 (10). However, we found that the TLR2 ligand Pam3CSK4 mimicked HKMT in regard to cathelicidin downregulation, although not quite as efficiently as HKMT. TLR4 triggering also resulted in cathelicidin downregulation and we could conclude that *M. tuberculosis* can downregulate cathelicidin expression in DC through signaling via TLR2 and most likely additional TLR. As for cathelicidin expression, different responses between monocytes/macrophages and DC to TLR2 signaling have been reported concerning MHC class II expression. Thus, whereas TLR2 signaling inhibits MHC class II expression in macrophages, it enhances MHC class II expression in DC (58). Interestingly, although the paradigm is that TLR2 signaling increases cathelicidin expression in human monocytes and macrophages (21, 22, 24), two papers have actually found that TLR2 signaling reduces cathelicidin expression in human monocytes in accordance with what we find in human DC (59, 60).

*M. tuberculosis* uses various strategies to evade antimicrobial mechanisms of macrophages, including inhibition of phagosome–lysosome fusion, antigen presentation, MHC class II expression, autophagy, and cytokine production (61). Interestingly, many of these immune evasion mechanisms are driven by TLR2 signaling (62–65). In the present study, we show that HKMT can use TLR2 signaling to strongly inhibit cathelicidin expression in DC, and as for autophagy (22, 24), we find that this attempt to evade the immune system by *M. tuberculosis* can be counteracted by vitamin D. Thus, although vitamin D reduces IFNγ production, we could conclude that vitamin D does not block Th1 development, and that Th1 cells produce significant amounts of IFNγ in the presence of vitamin D. At the same time, vitamin D is required to upregulate and counteract downregulation of cathelicidin by *M. tuberculosis*. We believe that these findings add to the understanding of the beneficial role of vitamin D in TB prevention and treatment.

## Ethics Statement

The study was approved by The Committees of Biomedical Research Ethics for the Capital Region in Denmark (H-16033682). Blood samples were obtained from healthy donors after obtaining informed, written consent in accordance with the Declarations of Helsinki principles for research involving human objects.

## Author Contributions

AR, MK, MH, DL, and TL performed the laboratory experiments. AR, MK, AW, NØ, CB, and CG conceived and designed the experiments. AR, MK, and CG analyzed the data and wrote the paper. All authors revised and approved the final manuscript.

## Conflict of Interest Statement

The authors declare that the research was conducted in the absence of any commercial or financial relationships that could be construed as a potential conflict of interest.
