# Supplementary figures and images for: Vitamin D Counteracts Mycobacterium tuberculosis-Induced Cathelicidin Downregulation in Dendritic Cells and Allows Th1 Differentiation and IFNγ Secretion
Source: Front Immunol. 2017 May 31;8:656. doi: 10.3389/fimmu.2017.00656 (PMC5450038; doi:10.3389/fimmu.2017.00656)

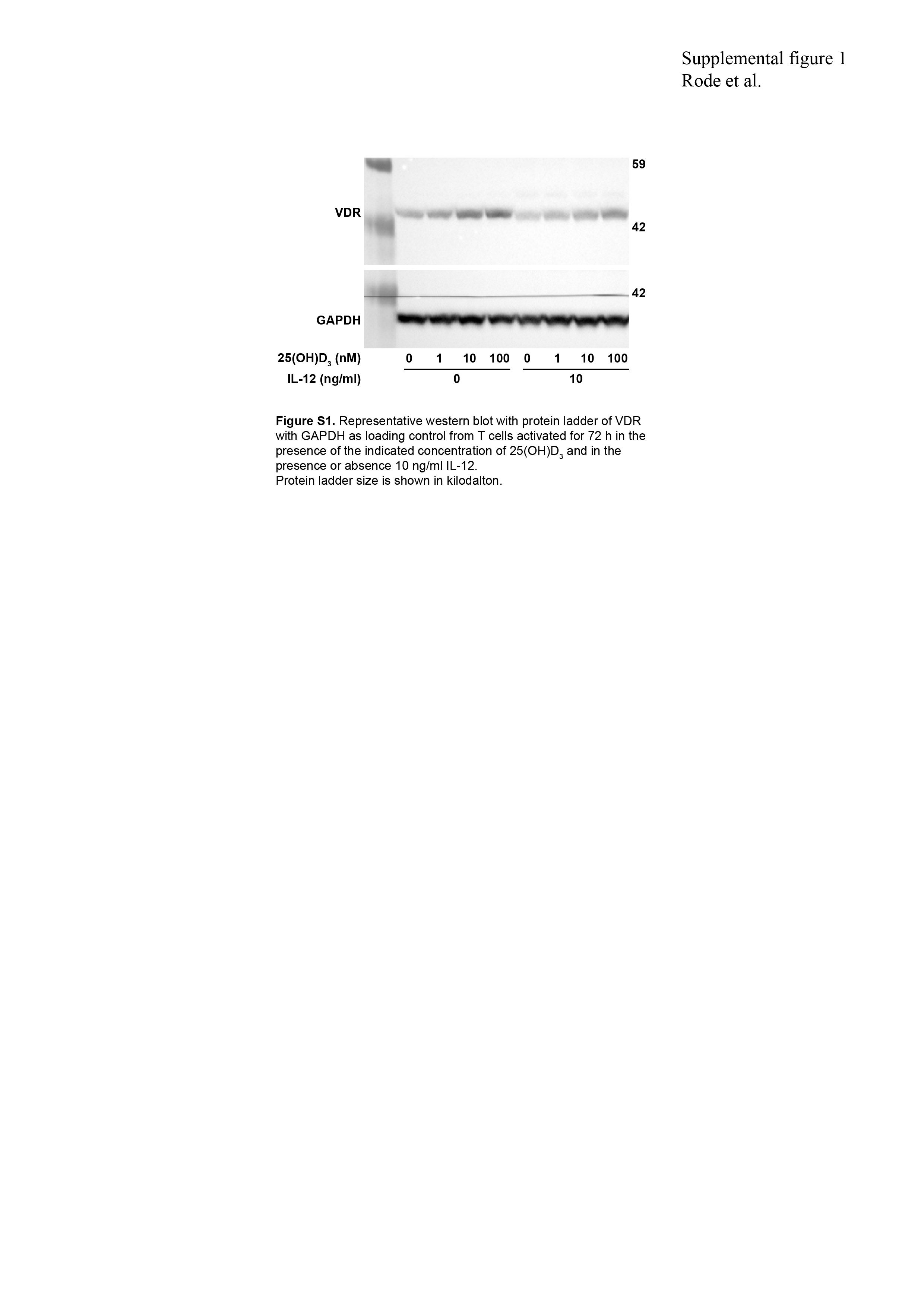

Supplement: Supplementary file 1 [file Image_1.JPEG]

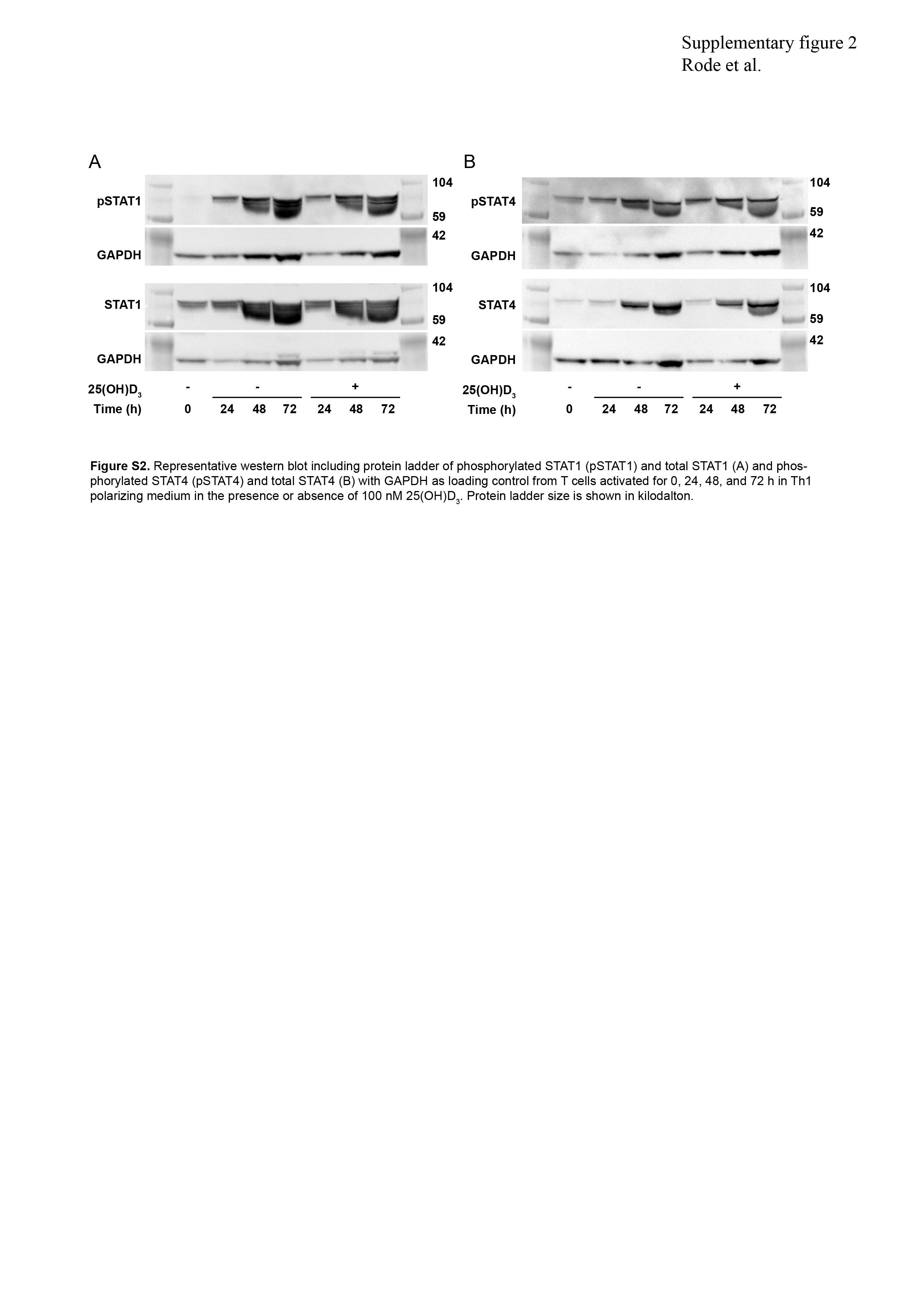

Supplement: Supplementary file 2 [file Image_2.JPEG]

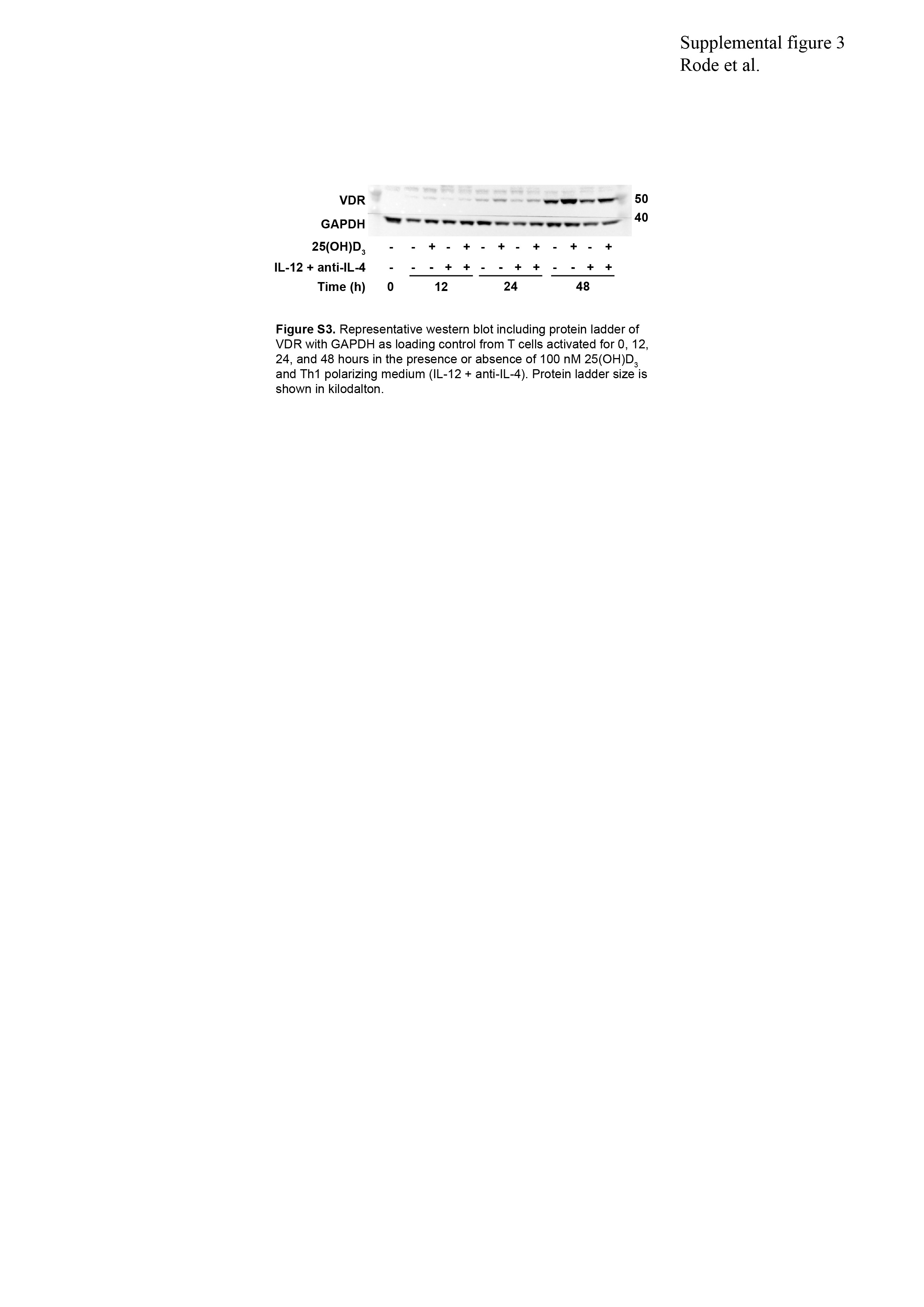

Supplement: Supplementary file 3 [file Image_3.JPEG]
